# Impact of Facebook and Newspaper Advertising on Sales: A Comparative Study of Online and Print Media

**DOI:** 10.1155/2021/5995008

**Published:** 2021-08-23

**Authors:** Yushan Lin, Zubair Ahmad, Wasswa Shafik, Saima K. Khosa, Zahra Almaspoor, Hassan Alsuhabi, Faheem Abbas

**Affiliations:** ^1^School of Marxism, Xi'an Jiaotong University, Xi'an, Shaanxi Province, China; ^2^Department of Statistics, Yazd University, P. O. Box 89175-741, Yazd, Iran; ^3^Computer Engineering Department, Intelligent Connectivity Research Laboratory, P. O. Box 89175-741, Yazd, Iran; ^4^Department of Statistics, Bahauddin Zakariya University, Multan, Pakistan; ^5^Department of Mathematics, Al-Qunfudah University College, Umm Al-Qura University, Mecca, Saudi Arabia; ^6^Department of Engineering Physics and Mathematics, Faculty of Engineering, Tanta University, 31521 Tanta, Egypt; ^7^Department of Mathematics, The University College in Jamoum, Umm Al-Qura University, Mecca 2064, Saudi Arabia

## Abstract

Marketing means the strategies and tactics an organization undertakes for attracting consumers to promote the buying or selling of a product or service. Active marketing is about receiving messages from potential buyers to create ways to influence their purchasing decisions. Advertising is one of the most prominent marketing strategies to promote products to consumers. It is well known that advertisement has a significant impact on the sale of certain goods or services. In this paper, we consider two mediums of advertisement, such as Facebook (which is an online medium) and Newspaper (which is a printed medium). We consider a dataset representing the advertising budget (in hundreds of US dollars) of an electronic company and the sales of that company. We apply the quantitative research approach, and the data which are used in this research are secondary data. For analysis purposes, we consider a statistical tool called simple linear regression modeling. To check the significance of the advertising on sale, definite statistical tests are applied. Based on the findings of this research, it is observed that advertising has a significant impact on sales. It is also showed that spending money on advertising through Facebook has better sales than newspapers. The finding of this research shows that the use of computer-based technologies and online mediums has a brighter future for advertising. Furthermore, a new statistical model is introduced using the *Z* family approach. The proposed model is very interesting and possesses heavy-tailed properties. Finally, the applicability of the proposed model is illustrated by considering the financial dataset.

## 1. Introduction

Marketing is a prominent approach to speaking to potential consumers about a particular brand. It helps to explore a specified product or service even more than the actual product or service does. The strategy of marketing can be divided into the four P's such as (i) product, (ii) place, (iii) price, and (iv) promotion. With the help of marketing, potential consumers can learn more detailed information about the brand. It has a positive role in influencing the customer's decision to buy a particular product or service; see Hajli and Lin [1], Chandel et al. [2], Wesley et al. [3], Johnston et al. [4], Wiese [5], and Focke et al. [6].

Considering the value of marketing in business survival and connectivity between customers and sales, it is expedient for businesses to engage in programs that may influence a customer's decision to purchase its products. This is where advertising and product management come in handy. Advertising is one of the most prominent ways of promoting and marking to make the consumers aware of the specified product or brand. It is obvious that advertisement has a positive impact on the sale of certain goods and services [7–13].

A useful advertising campaign uses a combination/mixture of different media to generate better excitement for a brand [14]. For instance, if the concerned product is related to a younger audience, then social/online media platforms, such as Twitter, Instagram, YouTube, and Facebook, might be the best medium to reach the target audience. Some other consumer groups (audience) may respond positively to other mediums such as radio, television, or print ads. The media such as television, radio, and newspaper (print media) make a huge impact on the population. It transforms our culture and becomes a tool for discovering new products and even learning. For a brief discussion about the importance of marketing and advertising, we refer to Dabija et al. [15], Sion [16], Sion [17], Winter [18], Balaban and Racz [19], Lee et al. [20], Clark [21], Mircica [22], and Pop et al. [23].

As a promotional strategy, advertising serves as a great tool in building product awareness and a potential consumer attitude, making a purchase decision in the end. In this article, we compare the impact of social media (Facebook) and print media (newspaper) on sales. Numerous statistical tools are available to compare the impact of advertising on sales. Among the available methods, we adopt the linear regression modeling approach to see the impact of advertising on sales. For this purpose, we use a statistical tool called hypothesis testing. The null hypothesis *H*_0_ and alternative hypothesis *H*_1_ can be formulated as follows: *H*_0_  = no significant relationship exists between Facebook advertising and sales vs. *H*_1_  = a significant relationship exists between Facebook advertising and sales. The same hypothesis will also be tested for the newspaper advertisement.

Furthermore, we know that the best description of data and prediction of the customer's behavior are very crucial for the business community to increase sales. In order to have the best description of the data related to the finance sector, numerous statistical models have been introduced in the literature. As we know that the financial datasets are usually skewed to the right, unimodal, hump-shaped, and possess a thick right tail; for details, see [24] and [25]. The heavy-tailed (HT) distributions have proven to be the best candidate models for modeling HT financial datasets.

Due to the importance of HT models in the finance sector, we introduce a new statistical model to provide the best fit to sales data. The proposed model is a new modification of the Weibull distribution and is introduced by adding only one additional parameter rather than adding two or three parameters. The new model has a closed-form distribution function (DF) which makes it easier to compute the mathematical properties and generate random numbers for simulation purposes. The proposed model is skewed to the right, unimodal, hump-shaped, and possesses a thick right tail. Furthermore, the HT behavior of the proposed model has also been proven mathematically in Section 7.

The rest of this paper is divided into 8 sections. The advertising mediums are discussed in Section 2. The methodology adopted for checking the impact of advertising mediums on sales is discussed in Section 3. The new model is introduced in Section 4. The HT properties of the new model are discussed in Section 5.  Section 6 deals with the statistical analysis. A brief discussion is provided in Section 7. Finally, the limitation, future research direction, and concluding comments are presented in Section 8.

## 2. Advertising Mediums

In the last couple of decades, we have seen that advertising had a clear and significant effect on sales. In this section, we discuss two advertising mediums, such as Facebook and newspaper.

### 2.1. Advertising through Facebook

Hadadi and Almsafir [26] presented some of the results that online advertising is the best option because it has a variety of different technologies, and people are encouraged to use social networks more than ever before. According to Duggan et al. [27], buying on social media platforms is different from regular online shopping. Women have a more positive attitude than men in terms of catalogs and stores, but this gender gap disappears when shopping online. Online advertising not only requires good contents but also needs to be distributed in areas frequently visited by customers and potential people [28].

**Figure 1:**
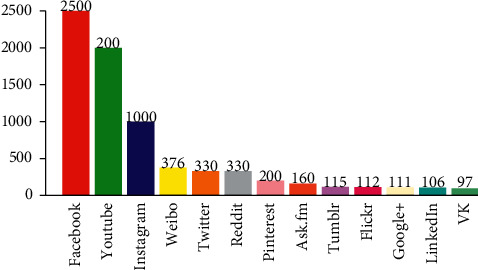
Users (in millions) of most popular social networks.

A social network is an online social networking site that has become part of everyday life. Social networking sites such as Facebook, MySpace, Friendster, Live Journal, LinkedIn, Cyworld, and Xiaonei are the popular social platforms that allow users to create a public profile and share texts and photos with others [29]. Among the most popular online advertising platforms, Facebook is the most important social networking site; see Figure 1.

It is known that around two billion people are using Facebook worldwide every day. Businesses are interested in exchanging information, marketing products, and interacting with current and potential customers to ensure a better understanding of the targeted customers. There is a need to understand the relationship between Facebook advertising and the benefits of this advertising. For a comprehensive study about the link between the benefits and advertising of Facebook, see [30].

### 2.2. Advertising through Newspaper

In advertising in media, the job of the advertiser is to have a clear understanding of the market for their product. The advertising budget is not always enough to allow year-round advertising. According to Bansal and Gupta [31], important factors such as cost, access, frequency, and direct audiences play a key role in choosing the best newspaper as their favorite advertising vehicle. Online advertising can be future research as an option for companies placing their ads. Newspapers with many readerships will have a strong demand for ads.

According to Simola et al. [32], print media spreads widely, and information flows faster to the people. These opportunities can provide high performance to influence people because they can find print media anywhere and flow faster than other ads. It is known not only for playing an effective role in informing people but also for changing people's think and shape people's attitudes.

The newspaper industry has undergone an unprecedented transformation and experiencing losses over the last two decades. One group of newspapers is concerned about the sale of real estate advertisers. Real estate is $11 billion worth of business each year, and newspapers have enjoyed a significant part of real estate advertising. As more consumers turn to online shopping, many companies are limiting their advertising in newspaper costs and directing it to online advertising.

According to Colussi and Rocha [33], newspapers are more affected by the economic downturn. Magazines suffer strongly from the economic recession. Therefore, online media has become a popular medium for advertising and marketing [34].

## 3. Methodology and Regression Analysis

In the field of management sciences, statistical methods are used quite effective to determine market trends. The secret to profitable advertising is to get the targeted point precisely and to use active marketing, social media, and accessibility programs. Statistical methods can help marketers to achieve the concerned goals.

In this section, we discuss a statistical application known as regression analysis, which plays a key role in determining market trends. Regression analysis is a very useful method in market research that assists the analyst in understanding that how the changes in the dependent variables are related to changes in independent variables; see [35] and [36].

In this paper, we limit our study to a simple linear regression modeling, only. Simple linear regression helps to measure the relationship between response variable, say *Y* (model output), and predictor, say *X* (model input). Mathematically, the simple linear regression model is defined by

(1) $$\begin{array}{c} Y=\alpha_{0}+\alpha_{1}X+\varepsilon , \end{array}$$

where *Y* is called the response variable, representing the outcome of the model that is what we are trying to predict, *X* is known as the predictor (also called independent) variable that helps in predicting the outcome, and *α*_0_ is a constant value called the intercept of the model and represents the starting point of the regression line. It is important to note that if *X* = 0 (means *X* does not affect *Y*), then *Y*=*α*_0_ · *α*_1_ is called the slope of the regression line, representing per unit changes in the outcome of the regression model, and *ε* is the residual error term having a mean value of 0. The error term includes all those factors which influence *Y*, but are not considered in the model.

Section 3.1 offers a regression analysis to predict the response variable (sale in this case) based on Facebook and newspaper advertisements.

### 3.1. Effects of Facebook and Newspaper Advertising on the Sales

In this section, we consider a linear regression technique to model the relationship between Facebook and newspaper advertising (taken as predictor variables) and sales. Mathematically, the linear regression model to explain the sales based on the Facebook advertising is given by

(2) $$\begin{array}{c} \text{Sales}=\alpha_{0}+\alpha_{1}\text{Facebook}+\varepsilon . \end{array}$$

After applying the regression technique, we observe that *α*_0_=12.769, interpreted as the predicted dollar sales for spending nothing on Facebook advertising. Henceforth, for a zero Facebook advertising budget, the expected sale is 12.769*∗*1000=$12769. The slope (regression coefficient) of the regression model provided in equation (2) is *α*_1_=0.176, indicating 0.176(0.176*∗*1000) units increment in the sales. Therefore, spending budget on Facebook advertising, the expected sale is 12.769+0.176*∗*1000=188.769, representing a sale of $188769. The fitted model corresponding to equation (2) is given by

(3) $$\begin{array}{c} \text{Sales}=12.7697+0.1765\text{Facebook}. \end{array}$$

Mathematically, the linear regression model to explain the sales based on the newspaper advertising is given by

(4) $$\begin{array}{c} \text{Sales}=\alpha_{0}+\alpha_{1}\text{Newspaper}+\varepsilon . \end{array}$$

For the model provided in equation (4), we observe that the values of *α*_0_ and *α*_1_ are given by 16.417 and 0.048, respectively. So, for no budget of newspaper advertising, the sale is expected to be 16.417*∗*1000=$16417. Whereas, the regression coefficient of the regression model is *α*_1_=0.048, indicating 48(0.048*∗*1000) units increment in the sales. Therefore, spending budget on newspaper advertising, the expected sale is 16.417+0.048*∗*1000=64.417, representing a sale of $64417. The fitted model corresponding to equation (4) is

(5) $$\begin{array}{c} \text{Sales}=16.417+0.048\text{Newspaper}. \end{array}$$

**Figure 2:**
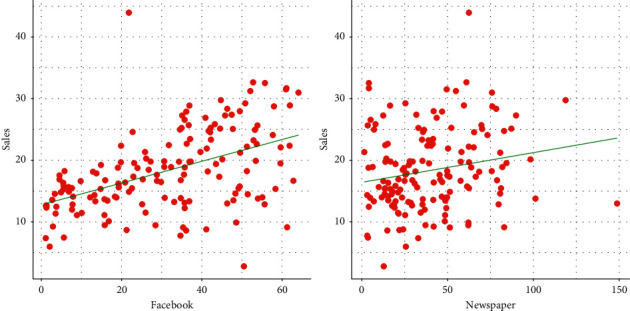
Relationship between Facebook and newspaper advertising and sales.

The relationship between Facebook and newspaper advertising and sales is displayed graphically in Figure 2. The plots, presented in Figure 2, indicate a positive linear relationship for advertising mediums. Therefore, spending more money on Facebook and newspaper advertising increases the sale.

### 3.2. Hypothesis Testing

To check the importance and significance of Facebook and newspaper advertising on sales, we adopt a statistical tool called hypothesis testing. The null hypothesis *H*_0_ and alternative hypothesis *H*_1_ can be formulated as follows: *H*_0_  = coefficients are equal to zero, i.e., no significant relationship exists between Facebook advertising and sales vs. *H*_1_  = coefficients are not equal to zero, i.e., a significant relationship exists between Facebook advertising and sales.

The standard errors are very useful in conducting hypothesis tests on the regression coefficients. It measures how much the coefficient estimates differ from the average value of *Y*. The standard error can also be used to calculate confidence intervals.

To test the null hypothesis, first, we have to find whether the estimates for *α*_0_ and *α*_1_ are sufficiently far from 0 such that *α*_0_ and *α*_1_ are nonzero. If the standard errors of the estimates of *α*_0_ and *α*_1_ are sufficiently small, then even small values of the estimates of *α*_0_ and *α*_1_ will provide sufficient evidence against the null hypothesis. We use the *t*-statistic to measure how far *α*_0_ and *α*_1_ are from zero.

As stated above, the standard error value quantifies the difference between the estimates and the average value of *Y.* In simple words, it measures the accuracy of estimates. The standard error is close to zero, representing a better estimate.

### 3.3. Statistical Testing

The *t*-statistic value indicates how far (in standard deviations) the coefficient estimate is from zero. A large value of the *t*-statistic provides evidence against the null hypothesis, indicating that a relationship exists between *X* and *Y*. The smaller the *p* value, the greater the likelihood of rejecting the null hypothesis. Generally, a *p* value of 0.05 is a standard cut-off point. A summary of the analysis is provided in Table 1.

From the results provided in Table 1, we can see that the value of the *t*-statistic (for both Facebook and Newspaper advertising) is far from zero and the *p* value is less than 0.05, indicating that the values of *α*_0_ and *α*_1_ are not equal to zero. Therefore, we reject the null hypothesis and conclude that there is a significant relationship between Facebook advertising and sales as well as newspaper advertising and sales.

The *F*-test statistic is another useful statistical tool used to detect the relationship between *Y* and *X*. The value of the *F*-test is away from zero, indicating the better regression model. As given in Table 2, the *p* values are less than 0.05, and the *F*-statistic is 43.66 and 5.729 for Facebook and newspaper advertising, respectively. Henceforth, using Facebook and newspaper advertising media as predictor variables to predict the sales indicates a better model.

### 3.4. Accuracy of the Model Fitting

The *R* square (*R*^2^) is the most important quantity for measuring regression model fit and varies between 0 and 1. It quantifies the linear relationship between *X* and *Y*. The value of *R*^2^ is close to 1, representing the better fit, and close to 0, representing the poor fit. For the case, advertising on Facebook, *R*^2^ is 0.227. So, spending budget on Facebook advertising, the sale can be increased up to 22.7%. Whereas, in the case of newspaper advertising, *R*^2^ is 0.037. So, spending budget on newspaper advertising, the sale can be increased up to 3.7%. From the above discussion, it is clear that Facebook advertising is more effective than newspaper advertising to increase sales.

### 3.5. Residuals

The residual standard error measures the quality of the regression fit. In this study, it represents the difference between the average amount of sales and the true regression line.

#### 3.5.1. Linearity of the Residuals

**Figure 3:**
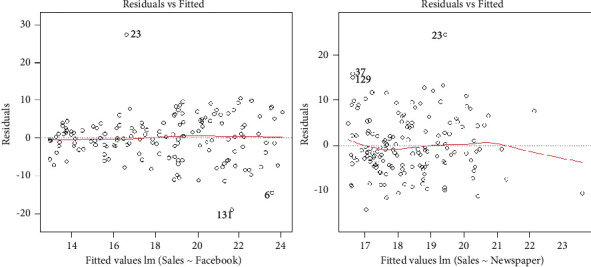
Residual plots of the Facebook and newspaper advertising.

The residuals are simply a measure of the error or the difference between the observed value of the response variable and predictor(s). The residuals vs. fitted plots of the Facebook and newspaper advertising are presented in Figure 3.

From the plots provided in Figure 3, the red line is almost lying near the residual value of 0 and is almost horizontal, and all the fitted values are scattered around them without a systematic relationship. Therefore, we conclude that the residuals are linearly related. The normality of the residual can be tested via two approaches such as the normality test and the graphical approach. Here, we use both approaches to check the normality of the residuals.

#### 3.5.2. Numerical Approach for Testing Normality of the Residuals

We perform the Shapiro–Wilk (S-W) normality test and Anderson–Darling (A-D) normality test to check the normality of the residuals. Under these two tests, the null and alternative hypothesis can be constructed as follows: *H*_0_ = the residuals are normally distributed vs. *H*_1_ = the residuals are not normally distributed. After performing the analysis, the values of the S-W and A-D normality tests along with the *p* values are provided in Table 3.

From, the results provided in Table 3, we can see that the *p* value is less than 0.05. Therefore, we conclude that the residuals are not normally distributed.

#### 3.5.3. Graphical Approach for Testing Normality of the Residuals

The *Q-Q* (quantile-quantile) plot is a graphical approach that helps us to determine whether the data collection is from a specific distribution such as normal, Weibull, or exponential. For example, if we conduct a statistical analysis that assumes that our variables are normally distributed, we can use a normal *Q-Q* graphical approach to test that assumption.

**Figure 4:**
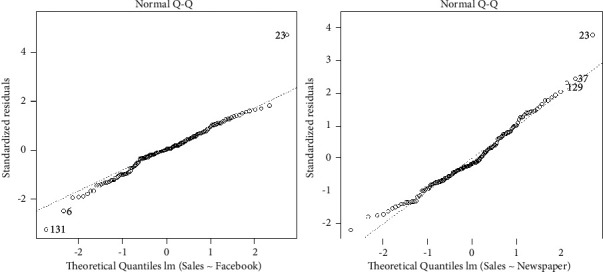
The residual vs fitted plots of the Facebook and newspaper advertising.

In fact, a *Q-Q* plot is a form of the scatter plot created by plotting two sets of quantiles against each other. If both sets of quantiles come from the same distribution, we should see all the points form a straight line. The *Q-Q* plots of the Facebook and newspaper are drawn in Figure 4, which shows the residuals are roughly linear related. Henceforth, we conclude that the normality is hardly met on residuals.

#### 3.5.4. Independence of the Residuals

In this section, we check the independence of residuals. We use the Durbin–Watson (D-W) test to check whether the residuals are independent or not. Under the D-W test, the null and alternative hypothesis can be constructed as follows: *H*_0_ = the residuals are not linearly related, i.e., residuals are independent vs. *H*_1_ = the residuals are linearly related, i.e., residuals are dependent. The numerical results are provided in Table 4.

From the results provided in Table 4, we can see that the *p* value is greater than 0.05. Therefore, we retain the null hypothesis, and we conclude that the residuals are not linearly related.

#### 3.5.5. Homoscedasticity of the Residuals

**Figure 5:**
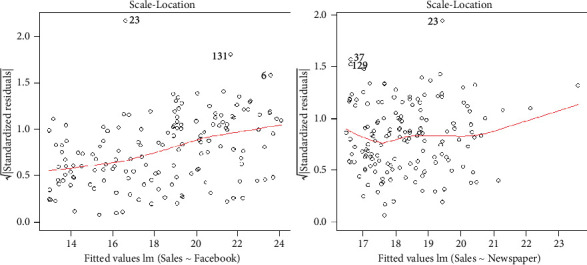
Scale-location plots of the Facebook and newspaper advertising.

Homoscedasticity is a useful assumption in the regression which states that the residuals are approximately constant for all values of the predictor variables. Violation of this assumption results in a large variance. From the scale-location plots provided in Figure 5, we can see that all the residuals are scattered leading to the fact that the homoscedasticity met on the residuals.

### 3.6. Influential Observations

**Figure 6:**
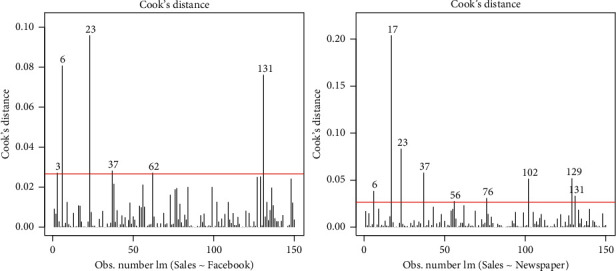
Cook's distance plots of the Facebook and newspaper advertising.

The influential observations have a significant effect on statistical analysis. In particular, in regression analysis, an influential observation is one whose deletion has a significant effect on the estimates. The influential observations in Facebook and newspaper advertising data using Cooke's distance approach are provided in Figure 6. The traditional cut-off of 4*/n* is used to identify influential observations. The numbers are crossing Cooke's distance line representing the influential observations.

## 4. The Proposed Distribution

The statistical distributions are used quite effectively for modeling real phenomena of nature. The development of new statistical distributions has received reasonable attention in the literature, especially, in actuarial and financial sciences. The new distributions are obtained either from the available distributions or derived from new families of distributions.

Recently, Ahmad et al. [37] proposed a *Z*-family of distributions by incorporating an additional parameter. The DF of the *Z*-family is defined by

(6) $$\begin{array}{c} G\left(t;\beta ,\Xi \right)=1-\left\{\frac{1-F\left(t;\Xi \right)}{\beta^{F\left(t;\Xi \right)}}\right\},\;\beta >0,t,\Xi \in \mathbb{R}, \end{array}$$

where *F*(*t*; Ξ) is a baseline distribution. The corresponding PDF (probability density function) is

(7) $$\begin{array}{c} g\left(t;\beta ,\Xi \right)=\frac{f\left(t;\Xi \right)}{\beta^{F\left(t;\Xi \right)}}\left\{1+\left(\log\;\beta \right)\left[1-F\left(t;\Xi \right)\right]\right\},\;t\in \mathbb{R}. \end{array}$$

Among the statistical distributions available in the literature, the HT distributions are more prominent for modeling data related to economics and finance. We further carry this area of research and introduce a new model to provide the best description of the financial data (sales).

A random variable say *T* has the inverse Weibull (IW) distribution if its DF with scale parameter *δ* > 0 and shape parameter *κ* > 0 are defined by

(8) $$\begin{array}{c} F\left(t;\Xi \right)=e^{-\delta t^{-\kappa }},\;t\geq 0, \end{array}$$

where Ξ=(*δ*, *κ*). The respective PDF is

(9) $$\begin{array}{c} f\left(t;\Xi \right)=\kappa \delta t^{-\kappa -1}e^{-\delta t^{-\kappa }},\;t>0. \end{array}$$

Here, we introduce a new three-parameter HT distribution called the *Z*-inverse Weibull (ZI-Weibull) distribution by using equation (8) in equation (6). The DF of the ZI-Weibull model is

(10) $$\begin{array}{c} G\left(t;\beta ,\Xi \right)=1-\left\{\frac{1-e^{-\delta t^{-\kappa }}}{\beta^{e^{-\delta t^{-\kappa }}}}\right\},\;t\geq 0,\beta ,\delta ,\kappa >0, \end{array}$$

with PDF

(11) $$\begin{array}{c} g\left(t;\beta ,\Xi \right)=\frac{\kappa \delta t^{-\kappa -1}e^{-\delta t^{-\kappa }}}{\beta^{e^{-\delta t^{-\kappa }}}}\left\{1+\left(\log\;\beta \right)\left(1-e^{-\delta t^{-\kappa }}\right)\right\},\;t>0. \end{array}$$

**Figure 7:**
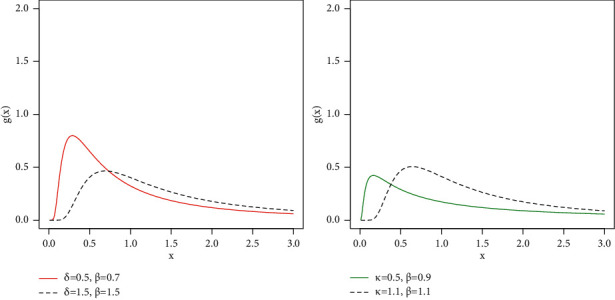
PDF plots of the ZI-Weibull distribution.

To see the effect of adding the additional parameter *β* to the IW distribution, we performed the following action, fixed the value of *κ*=1, and changed the values of (*δ*, *β*) and sketched the PDF plots (left panel of Figure 7). Next, we fixed the value of *δ*=1.2 and changed the values of (*κ*, *β*) and sketched the PDF plots (right panel of Figure 7). From the plots provided in Figure 7, we see that, as the value of the *β* increases, the ZI-Weibull distribution tends to a HT distribution.

The HT distributions are the ones, which satisfy the following condition:

(12) $$\begin{array}{c} \underset{t⟶\infty }{\lim\;}e^{pt}\left[1-G\left(t;\beta ,\Xi \right)\right]=\infty ,\;\forall p>0. \end{array}$$

An important class of HT distributions is the class of distributions possessing regularly varying behavior. A distribution is called regular varying if it obeys

(13) $$\begin{array}{c} \underset{t⟶\infty }{\lim}=\frac{1-G\left(ct;\beta ,\Xi \right)}{1-G\left(t;\beta ,\Xi \right)}=c^{-a},\;a\in \left[0,\infty \right], \end{array}$$

where the quantity *a* is known as an index of regular variation.

As in Figure 1, we showed that the ZI-Weibull is a HT distribution. In Section 5, we mathematically show that the ZI-Weibull distribution possesses the regularly varying tail behavior which is an important property of the HT distributions.

## 5. Heavy-Tailed and Regularly Varying Tail Behavior of the *Z*-Family

In this section, we deal with the heavy-tailed property as well as the regular variational behavior of the *Z*-family of distributions.

### 5.1. Heavy-Tailed Behavior

For a statistical distribution to obey the HT property, it is enough to prove that

(14) $$\begin{array}{c} \underset{t⟶\infty }{\lim}e^{pt}\left[1-G\left(t;\beta ,\Xi \right)\right]=\infty ,\;\forall p>0. \end{array}$$

Using equation (6) in equation (14), we observe

(15) $$\begin{array}{c} \underset{t⟶\infty }{\lim}e^{pt}\left[1-G\left(t;\beta ,\Xi \right)\right]=\underset{t⟶\infty }{\lim}\frac{e^{pt}\left[1-F\left(t;\Xi \right)\right]}{\beta^{F\left(t;\Xi \right)}}. \end{array}$$

Since *F*(*t*; Ξ) is a DF and continuous at *∞*, also if *t*⟶*∞*, then ∀*p* > 0. Thus, we have *e*^*p*·*∞*^=*∞*. Therefore, from equation (14), we see that

(16) $$\begin{array}{c} \underset{t⟶\infty }{\lim}e^{pt}\left[1-G\left(t;\beta ,\Xi \right)\right]=\infty \cdot 0, \end{array}$$

which is indeterminate. However, we have the following.

**Theorem 1 .**

If *F*(*t*; Ξ) is a HT model, then *G*(*t*; *β*, Ξ) is a HT model.

**Proof**

Since we have

(17) $$\begin{array}{c} \underset{t⟶\infty }{\lim}e^{pt}\left[1-G\left(t;\beta ,\Xi \right)\right]=\underset{t⟶\infty }{\lim}\frac{e^{pt}\left[1-F\left(t;\Xi \right)\right]}{\beta^{F\left(t;\Xi \right)}}, \end{array}$$

as we stated that *F*(*t*; Ξ) is a DF, so *F*(*t*; *∞*)=1; moreover,

(18) $$\begin{array}{c} \underset{t⟶\infty }{\lim}e^{pt}\left[1-F\left(t;\Xi \right)\right]=\infty . \end{array}$$

Thus,

(19) $$\begin{array}{c} \underset{t⟶\infty }{\lim}e^{pt}\left[1-G\left(t;\beta ,\Xi \right)\right]=\underset{t⟶\infty }{\lim}\frac{\infty }{\beta }=\infty . \end{array}$$

### 5.2. Regular Variational Result

The regular variational property is an important characteristic to identify the HT distributions. In terms of SF (survival function), we have the following.

**Theorem 2 .**

If $\bar{F}\left(t;\Xi \right)$ is a regular varying distribution, then $\bar{G}\left(t;\beta ,\Xi \right)$.

**Proof**

Suppose that $\left(\bar{F}\left(ct;\Xi \right)/\bar{F}\left(t;\Xi \right)\right)=g\left(c\right)$ is finite but nonzero for every *c* > 0. We observe that

(20) $$\begin{array}{c} \underset{t⟶\infty }{\lim}\frac{\bar{G}\left(ct;\beta ;\Xi \right)}{\bar{G}\left(t;\beta ,\Xi \right)}=\underset{t⟶\infty }{\lim}\frac{\bar{F}\left(ct;\Xi \right)}{\bar{F}\left(t;\Xi \right)}\times \frac{\beta^{F\left(t;\Xi \right)}}{\beta^{F\left(ct;\Xi \right)}},\\ \underset{t⟶\infty }{\lim}\frac{\bar{G}\left(ct;\beta ;\Xi \right)}{\bar{G}\left(t;\beta ,\Xi \right)}=\underset{t⟶\infty }{\lim}\frac{\bar{F}\left(ct;\Xi \right)}{\bar{F}\left(t;\Xi \right)}\times \frac{\beta^{e^{-\delta t^{-\kappa }}}}{\beta^{e^{-\delta c^{-\kappa_{t}-\kappa }}}}. \end{array}$$

Since *t*⟶*∞*, thus, we have

(21) $$\begin{array}{c} \underset{t⟶\infty }{\lim}\frac{\bar{G}\left(ct;\beta ;\Xi \right)}{\bar{G}\left(t;\beta ,\Xi \right)}=g\left(c\right)\times \frac{\beta }{\beta },\\ \underset{t⟶\infty }{\lim}\frac{\bar{G}\left(ct;\beta ;\Xi \right)}{\bar{G}\left(t;\beta ,\Xi \right)}=g\left(c\right), \end{array}$$

which is finite but nonzero for every *c* > 0; thus, *G*((*ct*; *β*; Ξ)) is the regular varying distribution. The function *g* is given by *g*(*c*)=*c*^*a*^, where *a* ∈ *ℝ* is a regular variation index, and *c* > 0.

### 5.3. Why the Regular Variational Result Can be True?

Assume that *T* has a power-law behavior; then, as per the definition of the HT distributions, we have

(22) $$\begin{array}{c} \bar{F}\left(t;\Xi \right)=\mathbb{P}\left(T>t\right)∼t^{-\alpha }. \end{array}$$

Now, according to Karamata's characterization theorem [38], we can write $\bar{G}\left(t;\beta ,\Xi \right)$ as

(23) $$\begin{array}{c} \bar{G}\left(t;\beta ,\Xi \right)=t^{-\alpha }L\left(t\right), \end{array}$$

where *L*(*t*) is slowly varying. As we have

(24) $$\begin{array}{c} \bar{G}\left(t;\beta ,\Xi \right)=\frac{\bar{F}\left(t;\Xi \right)}{\beta^{1-\bar{F}\left(t;\Xi \right)}}. \end{array}$$

Since $\bar{F}\left(t;\Xi \right)∼t^{-\alpha }$, we can write equation (4) as

(25) $$\begin{array}{c} \bar{G}\left(t;\beta ,\Xi \right)=\frac{t^{-\alpha }}{\beta^{1-t^{-\alpha }}},\\ \bar{G}\left(t;\beta ,\Xi \right)=t^{-\alpha }L\left(t\right), \end{array}$$

where *L*(*t*)=1/*β*^1−*t*^−*α*^^. So, if we can show that *L*(*t*) is a slowly varying function, then the variational result derived above is true. To prove *L*(*t*) is slowly varying, then we must show that

(26) $$\begin{array}{c} \underset{t⟶\infty }{\lim}\frac{L\left(at\right)}{L\left(t\right)}=1. \end{array}$$

So,

(27) $$\begin{array}{c} \frac{L\left(at\right)}{L\left(t\right)}=\frac{\beta^{1-t^{-\alpha }}}{\beta^{1-a^{-\alpha }t^{-\alpha }}}. \end{array}$$

From the above expression, we have

(28) $$\begin{array}{c} \underset{t⟶\infty }{\lim}\frac{L\left(at\right)}{L\left(t\right)}=1. \end{array}$$

## 6. Statistical Modeling

The prime interest of the development of the proposed distribution is to be applied for data analysis purposes. Here, this aspect is illustrated by considering the sales data. The data can be retrieved from https://data.world/datasets/sales. The comparison of ZI-Weibull distribution is done with the IW model. We estimate the unknown parameters of the fitted models using the maximum likelihood approach. The R-script with SANN algorithm is used to carry out the analysis.

For the sales dataset, the estimated values (with standard error provided in the parenthesis) of the ZI-Weibull parameters are $\overset{\wedge }{\kappa }=2.26517\left(0.11164\right)$, $\overset{\wedge }{\delta }=0.89510\left(0.08855\right)$, and $\overset{\wedge }{\beta }=0.38310\left(0.01509\right)$. Whereas, the estimated values of the IW distribution parameters are $\overset{\wedge }{\kappa }=1.96610\left(0.11164\right)$ and $\overset{\wedge }{\delta }=0.09733\left(0.15640\right)$.

**Figure 8:**
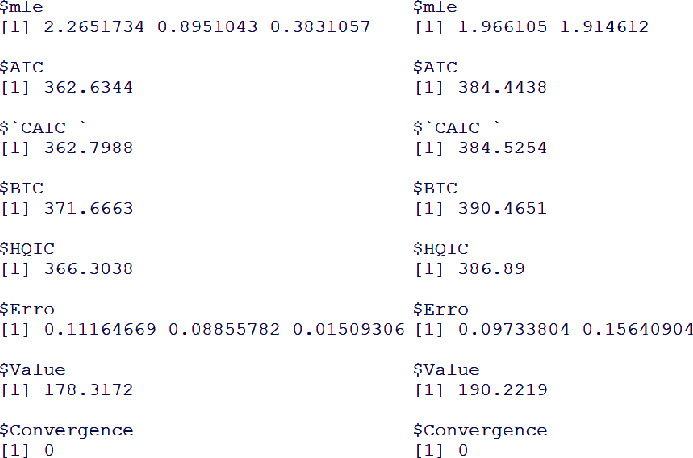
The MLEs and analytical measures of the fitted models.

Certain analytical measures such as AIC (Akaike information criterion), CAIC “Corrected Akaike information criterion), BIC (Bayesian information criterion), and HQIC (Hannan-Quinn information criterion) are considered to decide which distribution provides the best fit to the sales data. For the ZI-Weibull distribution, these measures are AIC=362.6344, CAIC=362.7988, BIC=371.6663, and HQIC=366.3038. Whereas, for the IW distribution, these measures are AIC=384.4438, CAIC=384.5254, BIC=390.4651, and HQIC=386.8900. These results are also shown in Figure 8.

Since the values of the analytical measures of the ZI-Weibull model are smaller than the IW distribution, therefore, we can conclude the proposed ZI-Weibull distribution may be a good candidate model for modeling financial and other related datasets.

**Figure 9:**
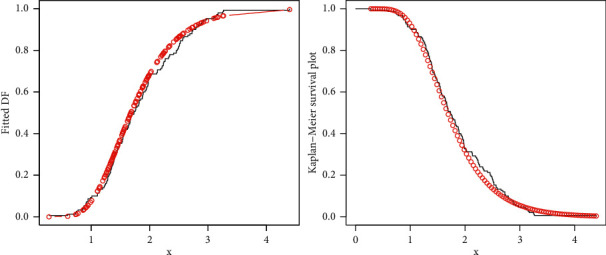
Fitted DF and Kaplan–Meier survival plots of the ZI-Weibull distribution for the sale data.

**Figure 10:**
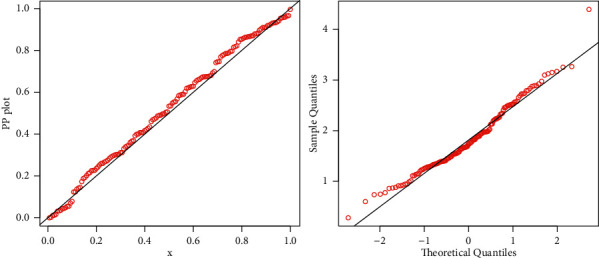
PP and QQ plots of the ZI-Weibull distribution for the sale data.

To support the numerical results provided in Figure 8, we sketched the plots of the fitted DF and Kaplan–Meier survival (see Figure 9), PP (probability-probability), and *QQ* (quantile-quantile) (see Figure 10). These plots graphically confirm the close fit of the ZI-Weibull distribution to the sales data.

## 7. Discussion

Marketing is a way of communication between the company and its customers. Advertising is a prominent tool for marketing to promote products to consumers. It is quite obvious that advertisement has a positive impact on the sale of certain goods or services. Among the possible advertising mediums, Facebook and newspaper are the most prominent ones. Among the online media platforms, Facebook is the fast growing one having two billion users worldwide everyday.

This study was focused on two main types of media advertising. However, each of these two major types has different popular kinds of advertising. The results of this research show that advertising mediums were linearly associated with sales. For advertising on Facebook, the value of *R*^2^ was 0.227 indicating a 22.7% increase in sales can be explained by Facebook. Whereas, for advertising in the newspaper, the value of *R*^2^ was 0.037 indicating a 3.7% increase in sales can be explained by a newspaper. Therefore, we can say that online advertising has a more significant impact on sales than print media.

From the value of *R*^2^, we can conclude that other factors may also affect sales, and the business community cannot increase sales by just applying Facebook and newspaper mediums for advertisement. Therefore, they need to improve their quality as well as services to attract more customers. The sale can be increased by utilizing more advertising platforms to convey awareness to the customers. This can be done by using more advertising mediums such as Youtube, Instagram, and Twitter, among others.

## 8. Concluding Remarks

In this work, we examined the relationship between advertising and sales. For this purpose, two advertising mediums such as Facebook advertising and newspaper advertising are considered. To test the significance of the advertising media, certain statistical tools such as *t*-statistic, *F*-statistic, and *R*^2^ quantity are considered. As per the findings of this research, there is a significant relationship between advertising and sales. It is observed that, by spending on advertising media, the expected sales can be four times higher than not spending on advertising. Furthermore, it is observed that advertising through online media is more effective than print media. We found that using online media for advertising has three times higher sales than print media. Based on the findings of this paper, it is recommended to use online media for advertising to increase the volume of sales. Finally, a new statistical model called the ZI-Weibull model is developed to provide the best description of the sales data. For illustrative purposes, the proposed model was applied to the financial data in comparison with the IW distribution. By considering certain analytical measures, it is observed that the proposed model is a good candidate model for modeling financial and other related datasets.

Despite the successful implementation of the proposed model to sales data, this study has some certain limitations. Some practical limitations of this study are given below:

  Since, the newspaper is used as an advertising medium in this paper, a serious limitation when it comes to targeting the customers/audience because the particular newspaper may not be available to the audience all the time.
  The lifespan of newspapers and magazines is very short as people have a tendency to throw them or keep them aside after one day of reading.
  Facebook is another medium of advertising considered in this work which can be accessed through the Internet easily. However, the Internet facilities may not be available everywhere and most of the time, and the advertisement might get lost.
  In this work, only two mediums of advertisement are considered. However, other mediums might also have a great impact on sales such as YouTube, Twitter, and Instagram, among others.
  Due to the complicated form of the PDF of the proposed model, the maximum likelihood estimators do not have a simple form. Therefore, an iteration procedure such as the Newton–Raphson method must be used via computer software to obtain the numerical values of the model parameters.

As we stated above that only two mediums of advertisement are considered in this paper. In the future, we are motivated to consider other mediums of advertisement to see their impact on sales. These mediums include the following.

Effect of YouTube advertising on sales: we are motivated to consider YouTube as an advertising medium to check its impact on sales. Mathematically, the linear regression model to explain the sales based on the YouTube advertising is given by
  (29) $$\begin{array}{c} \text{Sales}=\alpha_{o}+\alpha_{1}\text{YouTube}+\varepsilon . \end{array}$$
Effect of Instagram advertising on sales: another possible advertising medium is Instagram. Mathematically, the linear regression model to explain the sales based on the Instagram advertising is given by
  (30) $$\begin{array}{c} \text{Sales}=\alpha_{o}+\alpha_{1}\text{Instagram}+\varepsilon . \end{array}$$
Effect of Twitter advertising on sales: another interesting and fastly growing advertising medium is Twitter. Mathematically, the linear regression model to explain the sales based on the Twitter advertising is given by
  (31) $$\begin{array}{c} \text{Sales}=\alpha_{o}+\alpha_{1}\text{Twitter}+\varepsilon . \end{array}$$

## Figures and Tables

**Table 1:** Simple linear regression analysis.

AM	Coefficients	Estimated values	Standard error	*t*-statistic	*p* value
Facebook	*α*_0_	12.769	0.968	13.190	2*e* − 16
	*α*_1_	0.176	0.026	6.607	2*e* − 16
Newspaper	*α*_0_	16.417	0.962	17.063	2*e* − 16
	*α*_1_	0.048	0.020	2.394	0.017

**Table 2:** Simple linear regression analysis.

AM	*R* ^2^	Adjusted *R*^2^	*F*-statistic	*p* value	Degree of freedom
Facebook	0.227	0.222	43.66	6.6*e* − 10	1 and 148
Newspaper	0.037	0.030	5.729	0.017	1 and 148

**Table 3:** Normality tests of the data.

AM	Normality tests	Normality tests values	*p* value
Facebook	S-W	0.9654	0.00079
	A-D	0.6687	0.07924
Newspaper	S-W	0.9761	0.01027
	A-D	0.99061	0.01263

**Table 4:** Normality tests of the data.

AM	D-W test	Lag	Autocorrelation	*p* value
Facebook	1.893	1	0.044	0.490
Newspaper	1.990	1	−0.002	0.926

## Data Availability

All the datasets used in this paper are available from the corresponding author upon request.
